# Application of artificial intelligence‐based magnetic resonance imaging in diagnosis of cerebral small vessel disease

**DOI:** 10.1111/cns.14841

**Published:** 2024-07-24

**Authors:** Xiaofei Hu, Li Liu, Ming Xiong, Jie Lu

**Affiliations:** ^1^ Xuanwu Hospital Capital Medical University Beijing China; ^2^ Department of Nuclear Medicine, Southwest Hospital Third Military Medical University (Army Medical University) Chongqing China; ^3^ Department of Digital Medicine, School of Biomedical Engineering and Medical Imaging Third Military Medical University (Army Medical University) Chongqing China

**Keywords:** artificial intelligence, cerebral small vessel disease, deep learning, magnetic resonance imaging, review

## Abstract

Cerebral small vessel disease (CSVD) is an important cause of stroke, cognitive impairment, and other diseases, and its early quantitative evaluation can significantly improve patient prognosis. Magnetic resonance imaging (MRI) is an important method to evaluate the occurrence, development, and severity of CSVD. However, the diagnostic process lacks quantitative evaluation criteria and is limited by experience, which may easily lead to missed diagnoses and misdiagnoses. With the development of artificial intelligence technology based on deep learning, the extraction of high‐dimensional features in imaging can assist doctors in clinical decision‐making, and it has been widely used in brain function and mental disorders, and cardiovascular and cerebrovascular diseases. This paper summarizes the global research results in recent years and briefly describes the application of deep learning in evaluating CSVD signs in MRI imaging, including recent small subcortical infarcts, lacunes of presumed vascular origin, vascular white matter hyperintensity, enlarged perivascular spaces, cerebral microbleeds, brain atrophy, cortical superficial siderosis, and cortical cerebral microinfarct.

## Graphical abstract

Recent advancements in artificial intelligence, particularly deep learning, have revolutionized the detection and evaluation of cerebral small vessel disease (CSVD) through magnetic resonance imaging (MRI). This study demonstrates how deep learning can extract high‐dimensional imaging features, improving the quantitative assessment of CSVD, and leading to earlier diagnosis and better patient outcomes.

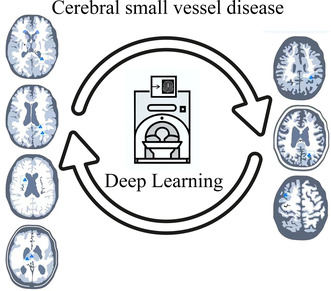

## 1 INTRODUCTION

Cerebral small vessel disease (CSVD) refers to a range of clinical, imaging, and pathological syndromes caused by various etiologies that affect the cerebral small arteries, brain venules, and capillaries. It is a pathological consequence of brain small vessel disease that affects the brain parenchyma, and the scope of small cerebral vessels includes small cerebral arteries (100–400 μm diameter), arterioles (40–100 μm diameter), capillaries, venules, and small veins.^1^ The incidence of CSVD increases with age, and there is no significant gender difference. CSVD accounts for 25% of strokes, and its incidence in recurrent strokes is as high as 50%.^2^ Additionally, 45% of dementia cases are caused by CSVD.^3^ In low‐ and middle‐income countries, such as China, the prevalence rates in the community of moderate‐to‐severe white matter hyperintensity (WMH), stroke, and dementia are 20.5%, 40.5%, and 58.4%, respectively. The prevalence rates of cerebral microbleeds (CMBs) in the community and stroke populations are 10.7% and 22.4%, respectively.^4^ The prevalence rate of enlarged perivascular spaces (PVS) in the community population is 25.0%, significantly higher than those in Western countries.^5^ Because nerve cell damage is irreversible and has high disability and mortality rates, early detection, diagnosis, and intervention are the key factors affecting the treatment of neurological diseases.^6^

**FIGURE 1:**
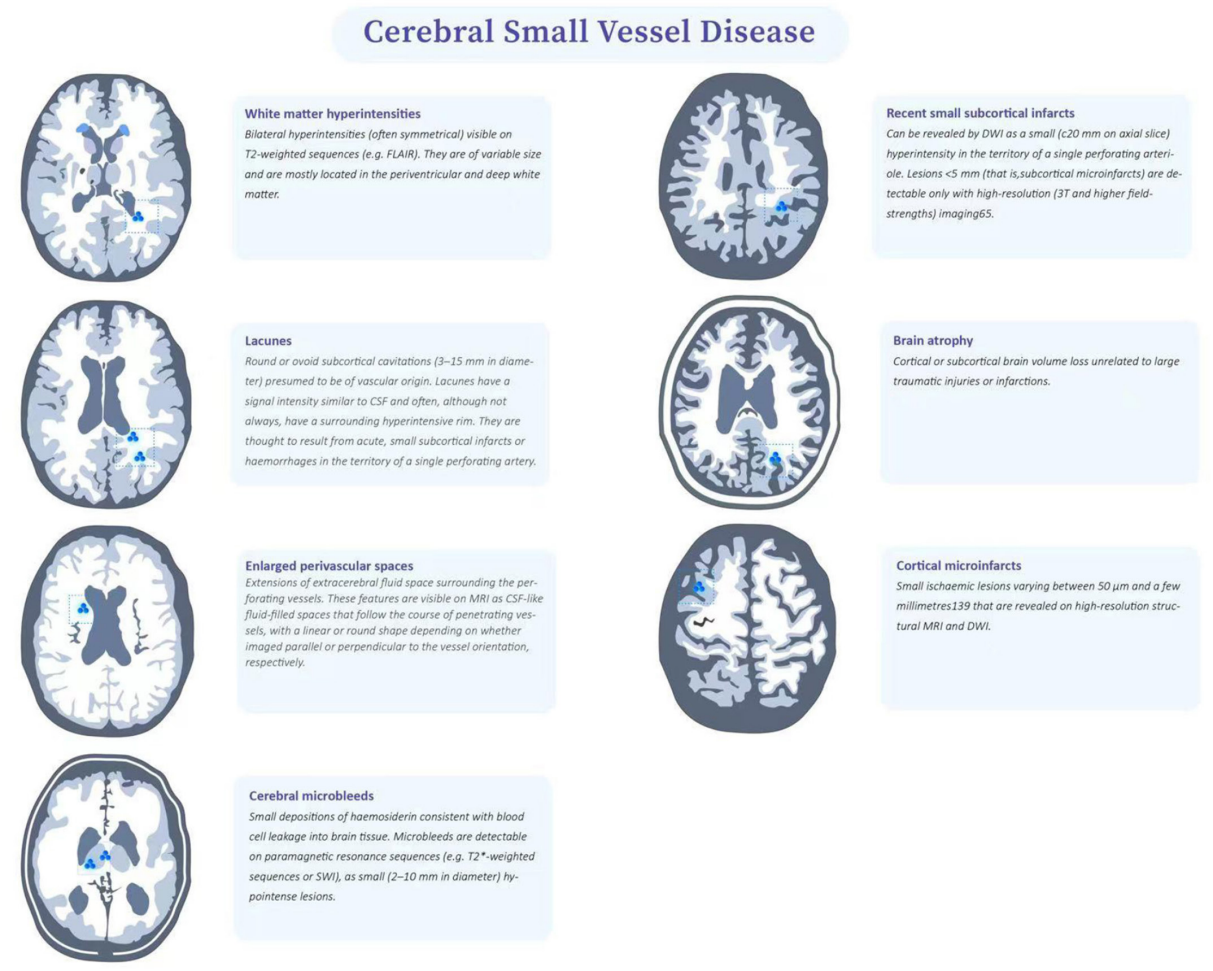
Typical imaging features of CSVD in MRI. Reprinted with permission from Wang et al.^54^

At present, the diagnostic methods for CSVD include imaging examination, clinical scale evaluation, blood pressure evaluation, laboratory examination, and genetic evaluation. Among these, magnetic resonance imaging (MRI) is the most important and widely used diagnostic modality for CSVD.^7^ Multimodal MRI combines different imaging techniques to provide information on anatomy, function, metabolism, and other aspects of brain tissue in patients with CSVD and to evaluate changes in the brain tissue in various periods caused by CSVD, as well as their relationship with clinical symptoms. It is of great significance to assess the occurrence, development, and severity of CSVD.^8^ The Standards for Reporting Vascular Changes on Neuroimaging (STRIVE) for small vessel disease of the brain imaging research, provided a unified understanding and standardized terminology for the MRI findings of CSVD,^9^ which established a foundation for the standardization and clinical collaboration of international CSVD imaging research.^9^, ^10^ (Figure 1). The updated STRIVE‐2 criteria expanded the typical MRI features of CSVD to include eight elements including recent small subcortical infarcts (RSSI), lacunes of presumed vascular origin, WMH, PVS, CMBs, cortical superficial siderosis, brain atrophy, and cortical cerebral microinfarct.^11^

Traditional diagnosis of CSVD is based on a comprehensive evaluation of the typical MRI signs of CVSD to obtain the total load score and provide the corresponding clinical diagnosis decision, such as evaluating the degree of brain atrophy using T1‐weighted MRI images and counting vascular lacunar foci based on T1‐weighted MRI and T2‐fluid‐attenuated inversion recovery (FLAIR) images. Moreover, for the evaluation of WMH, in addition to using different sequences, such as T1‐weighted, T2‐weighted, and T2‐FLAIR, it is necessary to use the Fazekas scale, which is a standard for grading WMH on imaging.^12^, ^13^ It evaluates paraventricular and deep WMH separately and finally judges the WMH level according to the sum of the scores. Unfortunately, despite its significant impact, accurate diagnosis of CSVD remains a challenge due to variations in imaging protocols, inadequate quantitative metrics, and clinician bias. Additionally, the interpretation of neuroimaging data can be subjective and highly dependent on the clinician's experience and training. Therefore, the accurate diagnosis of CSVD is a challenge, and a quantitative method for rapid and precise diagnosis is urgently needed.

To address these challenges, there is a growing need for the development of artificial intelligence (AI) tools that can assist with the analysis and interpretation of neuroimaging data of CVSD. Deep learning based on convolutional neural networks (CNNs) is important in AI and is different from traditional AI algorithms, such as the support vector machine, random forest, and Bayesian algorithms. This approach can self‐learn, summarize, and introduce existing data to generate an intelligent system with the support of big data and graphics processing unit (GPU).^14^, ^15^ It is an important extension of the traditional neural network and has been widely used in clinical auxiliary screening, diagnosis, classification, treatment decision‐making and guidance, and efficacy evaluation of major diseases, such as brain function and mental disorders, cardiovascular, and cerebrovascular diseases.^16^, ^17^ AI can also aid in the analysis of large datasets, allowing for the identification of new imaging biomarkers of cerebrovascular diseases and improved understanding of the disease's progression and underlying mechanisms.

At present, deep learning models are developed for object segmentation, classification, and detection of MRI features of CSVD. However, there are also challenges associated with the development and implementation of AI tools in neuroimaging of CSVD, including issues related to data quality, algorithm development, and regulatory compliance, and a considerable gap exists between its current researches and clinical application. Therefore, the primary purposes of this review are to clarify and summarize the application of deep learning to evaluate CSVD in MRI, as well as opportunities and challenges.

### 1.1 Database search strategy

Articles for the literature review were identified by searching in The Web of Science Core Collection (WoSCC) database. To cover studies that discussed the AI on CSVD, the search terms used were the following: TS = (“Cerebral Small Vessel Diseases” OR “Recent small subcortical infarct” OR “Lacune” OR “White matter hyperintensity” OR “Perivascular space” OR “Cerebral microbleed” OR “Cortical superficial siderosis” OR “Brain atrophy” OR “Cortical cerebral microinfarct”) AND TS = (((automated OR intelligent) NEAR/1 (classification OR diagnosis OR segment* OR detect*)) OR “artificial intelligence” OR “deep learning” OR “convolutional neural network*” OR “machine learning” OR “CNNs” OR “artificial neural network*” OR “computer‐aided” OR “Bayes* network” OR “computer‐assisted” OR (deep NEAR/1 network*) OR “U‐net”) AND TS = (“Magnetic resonance imaging” OR “MRI”). Original articles published between January 2002 and September 2022 were retrieved in this nonsystematic review. After reviewing the title, abstract, and keywords, those that were potentially related to the applications of AI‐based MRI in the diagnosis of CSVD were included.

**FIGURE 2:**
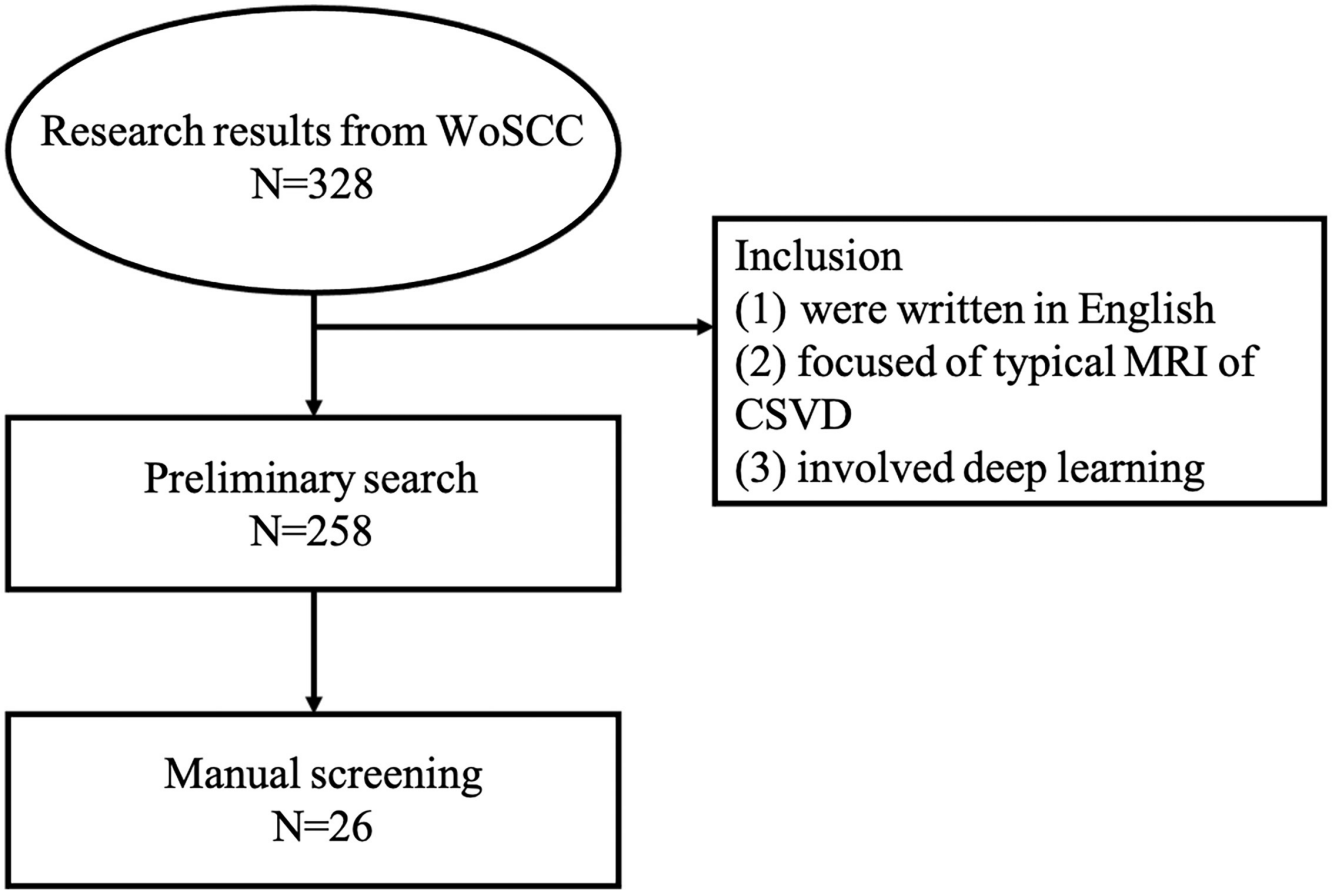
Flowchart of the search process in the study.

According to our research area, which focuses on the applications of AI in CSVD, we designed the following search items: the papers for analysis were restricted to those that (1) were written in English, (2) focused on typical MRI features (RSSI, lacunes of presumed vascular origin, WMH, PVS, CMBs, cortical superficial siderosis, brain atrophy, and cortical cerebral microinfarct) of CSVD, (3) involved deep learning. After the preliminary search, 258 papers were included, and then we conducted further manual screening. In the manual screening process, all papers are divided into relevant, uncertain, and excluded. Papers marked as unsure were screened by three of the authors (XH, LL, and MX) and discussed to determine whether they were included. According to the screening criteria, 233 papers were excluded because they neither focused on the field. Finally, 26 papers were included in our study (Table 1, Figure 2).

**TABLE 1:** Studies on the applications of AI in MRI imaging signs of CSVD.

Typical MRI features of CSVD	Number of patients	References
RSSI	1010	Duan et al., 2020^20^
Lacune of presumed vascular origin	2005	Duan et al., 2020^20^; Sudre et al., 2023^22^
WMH	5933	Duan et al., 2020^20^; Schirmer et al., 2019^34^; Hong et al., 2020^33^; Jiang et al., 2020^30^; Zhou et al., 2020^27^; Liang et al., 2021^31^; Park et al., 2021^29^; Rieu and Kim, 2021^26^; Sundaresan et al., 2021^28^; Sundaresan et al., 2021^32^; Zhang et al., 2022^36^; Rachmadi et al., 2020^37^
CMBs	40,217	Duan et al., 2020^20^; Rashid et al., 2021^38^; Sudre et al., 2023^22^
PVS	2486	Han et al., 2021^55^; Dubost et al., 2019^41^; Sudre et al., 2023^22^
Brain atrophy	3580	Bernal et al., 2021^50^; Pantoni, 2010^6^; Jin et al., 2021^49^

Abbreviations: CMBs, cerebral microbleeds; PVS, enlarged perivascular spaces; RSSI, recent small subcortical infarcts; WMH, vascular white matter hyperintensity.

## 2 INTRODUCTION TO ARTIFICIAL INTELLIGENCE ALGORITHMS

**FIGURE 3:**
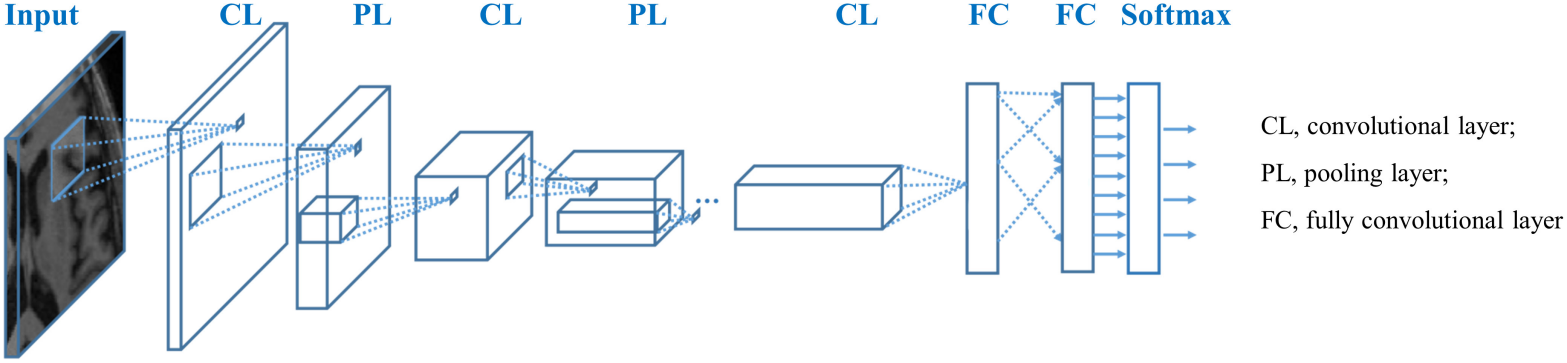
Typical three‐layer convolutional neural network.

A CNN in AI is a mathematical model that imitates the structure and function of a biological neural network, and it is also an adaptive computing model.^15^ Among them, CNN is the most successful AI for image analysis, and its applications include organ or lesion segmentation, anomaly detection, disease classification, and computer‐aided diagnosis.^18^, ^19^ A typical CNN structure is composed of numerous neurons, mainly including a convolutional layer, pooling layer, activation layer, and a fully connected layer (Figure 3). The convolutional layer is the core layer for building the CNN, primarily for feature extraction, to obtain the feature representation in the input image. The arrangement of the neurons in the convolutional layer constitutes a feature map. The pooling layer filters the features in the receptive field for the most representative feature extraction and effectively reduces the output feature scale, thereby reducing the number of parameters required by the model. Several convolutional and pooling layers are stacked to form a deep model to retrieve high‐level representations. Fully connected layers are used to interpret these features and perform high‐level inference functions. The workflow of CNN includes three parts: input, calculation, and output. In the medical image processing task, the input part is mainly digital medical images, and the calculation part is composed of numerous neurons, which are responsible for calculating the input medical image. This is actually a feature extraction process. In the output part, different results are obtained according to different tasks.

Depending on the different targets, intelligent analysis methods for medical images include network models such as target segmentation, classification, and detection. A segmentation network is mainly used to identify the contour or internal voxel set of regions of interest in medical images, such as organs and lesions. Organ or substructure segmentation based on medical images can provide quantitative results of clinical information, such as volume and shape for clinical diagnosis and is a key step in supporting disease diagnosis, surgical planning, prognostic assessment, and follow‐up. Typical segmentation algorithms include fully convolutional neural networks (FCNs), deep‐supervised networks (DSNs), and U‐Net networks. The classification network is mainly an algorithm that uses medical images to diagnose abnormal lesions in the organism and quantifies and grades the severity of the lesion area. Classic classification algorithms include VGGNet, GoogleNet, ResNet, and DenseNet, most of which were developed and designed based on natural image datasets. An object detection network refers to a network model that locates anatomical structures, such as organs and lesions, from images. Currently, mainstream detection algorithms include R‐CNN, Fast R‐CNN, Mask‐RCNN, and YOLO. The basic principle is to use a CNN to extract deep features from the image, then use the detection frame to establish a set of candidate regions, and finally build a classification network to classify the candidate regions and return the detection frame to achieve high target detection accuracy and speed.

## 3 APPLICATION OF ARTIFICIAL INTELLIGENCE IN MRI IMAGING SIGNS OF CSVD

### 3.1 Application of AI in RSSI

RSSI refers to a recent infarction in the area where the blood supply of a perforating artery is confirmed by neuroimaging and is accompanied by imaging features consistent with the recent brain injury. RSSIs are mainly distributed in the blood supply area of the perforating artery, mainly in the basal nerve nucleus area, the center of the semi‐oval, the radial crown, the brain stem, and other parts. The diameter in the axial plane is generally ≤20 mm and the diameter in the coronal or sagittal plane can exceed 20 mm. RSSIs appear as low signals on T1‐weighted images and high signals on T2‐weighted and T2‐FLAIR images, and the clinical diagnosis is based on a high diffusion‐weighted imaging (DWI) signal.

The intelligent diagnosis of RSSI segments the region of interest using a segmentation network to quantitatively evaluate the RSSI. Duan et al.^20^ obtained 1010 DWI (b = 1000) images from 1010 patients with subcortical infarction and constructed a subcutaneous infarction segmentation model based on CNN. The results showed that the segmentation result of the model (Dice = 0.728) was better than those of the four junior clinicians (<10 years) (Dice = 0.615, 0.690, 0.717, 0.747, respectively).

### 3.2 Application of AI in lacune of presumed vascular origin

Lacunes of presumed vascular origin are defined as cerebrospinal fluid (CSF)‐filled cavities that appear as circular or oval‐shaped lesions located in the subcortical part of the brain, typically between 3 and 15 mm in diameter. They are usually found in the basal ganglia or centrum semiovale regions. However, they may also occur in other locations such as the cortical subregions of the frontal, parietal, temporal, and occipital lobes, as well as in the cerebellum. When the scanning plane is perpendicular to the vascular section, typically exhibits low signal intensity on T1‐weighted imaging, high signal intensity on T2‐weighted imaging, a high signal intensity rim surrounding a central low signal or nodular high signal on T2‐FLAIR, and an iso‐ or low signal intensity on DWI and high signal intensity on apparent diffusion coefficient (ADC) maps.

Putative vascular lacunar foci are the main imaging characteristics of CSVD and are associated with a higher risk of stroke and dementia.^9^ Therefore, the detection of lacunes of presumed vascular origin is of great significance for elucidating the pathogenesis of neurodegenerative diseases, and the intelligent diagnosis of lacunes of presumed vascular origin uses segmentation, detection, or classification networks to determine whether the lesion exists in the brain, and the location and count of the lesion (such as in the basal ganglia or thalamus) based on the segmentation and detection results are definite. Recently, 3D CNN has been used to automatically detect and segment CSVD‐related vascular lacunar foci on MRI. Mohsen et al.^21^ proposed a deep multi‐scale location‐aware algorithm based on a 3D CNN for the automated detection of lacunes of presumed vascular origin. Trained and tested on a large dataset of 1075 cases, the model achieved a sensitivity of 0.974 with 0.13 false positives per slice. The detection performance was comparable to that of four experienced human observers. Duan et al.^20^ collected T1‐weighted and T2‐FLAIR data from 824 patients with lacunes of presumed vascular origin and constructed a CNN model for the segmentation of vascularized lacunar foci. In the test dataset of the 30 cases, the Dice value of the model was 0.496. This result is superior to the manual segmentation results of six experienced clinicians (Dice = 0.423), and the segmentation time is approximately 1/100 of the physician's average delineation time. In the context of the VALDO challenge, six teams focused on the detection and segmentation of lacunes, employing a dataset composed of T1‐weighted, T2‐weighted, and FLAIR images from 106 patients, identifying a total of 268 lesions.^22^ The methodologies applied included a combination of Mask R‐CNN and UNet, MaskRetinaNet, and UNet. The performance metrics revealed a detection F1 score of 28.57, and a mean Dice score for segmentation of 45.75.

The majority of the proposed methods were trained as pure segmentation solutions and a few teams submitted a detection+segmentation solution based on Mask‐RCNN^23^ or Mask Retina net.^24^ Across all tasks, when a deep learning solution was proposed, the UNet architecture was the most common choice. For all three tasks, the time required to process a case and the GPU memory requirements varied greatly. For Task 2—Microbleeds for instance duration ranged from less than 1 minute to 45.8 min and memory consumption of 2.4–43 GB (allowing for memory flooding). In terms of the methodology for uncertainty assessments in Task 3—Lacunes, the two teams submitting methods to all three tasks did not provide any uncertainty map. Cross‐dataset variability performance varied greatly across datasets. Most deep learning solutions are described using a UNet style architecture at one point of their pipeline either as the main network for one‐stage methods or for the segmentation component for multi‐stage solutions. Performance varied greatly across these teams. This could potentially be explained by the choice of input data, the dimensionality, or the framework chosen. In the context of microbleeds, using 3D information may be particularly relevant to avoid mimics. In terms of dataset origins, performance was generally higher for the dataset with the highest resolution. Lacunes with more heterogeneous shapes, performance appeared quite poor on both detection and segmentation metrics. The performance was higher on tasks for which the variability in element appearance was lower (EPVS with linear shapes and microbleeds with spherical shapes).

### 3.3 Application of AI in WMH

A WMH is a punctuated or patchy lesion around the ventricle or in the center of the semiovale, which appears hyperintense on T2‐FLAIR images and hypointense on T1‐weighted imaging images. At present, the pathogenesis of WMH is relatively complex, and the signal of WMH in different parts has different pathological characteristics, which cannot be explained by ischemia or single pathogenesis. With the continuous development of imaging technology, numerous studies have shown that WMH is related to some important clinical symptoms and risk factors and is an important marker of stroke, dementia, cognitive decline, increased risk of depression, and impaired gait and activity.^25^ Therefore, WMH has gradually begun to receive attention.

**FIGURE 4:**
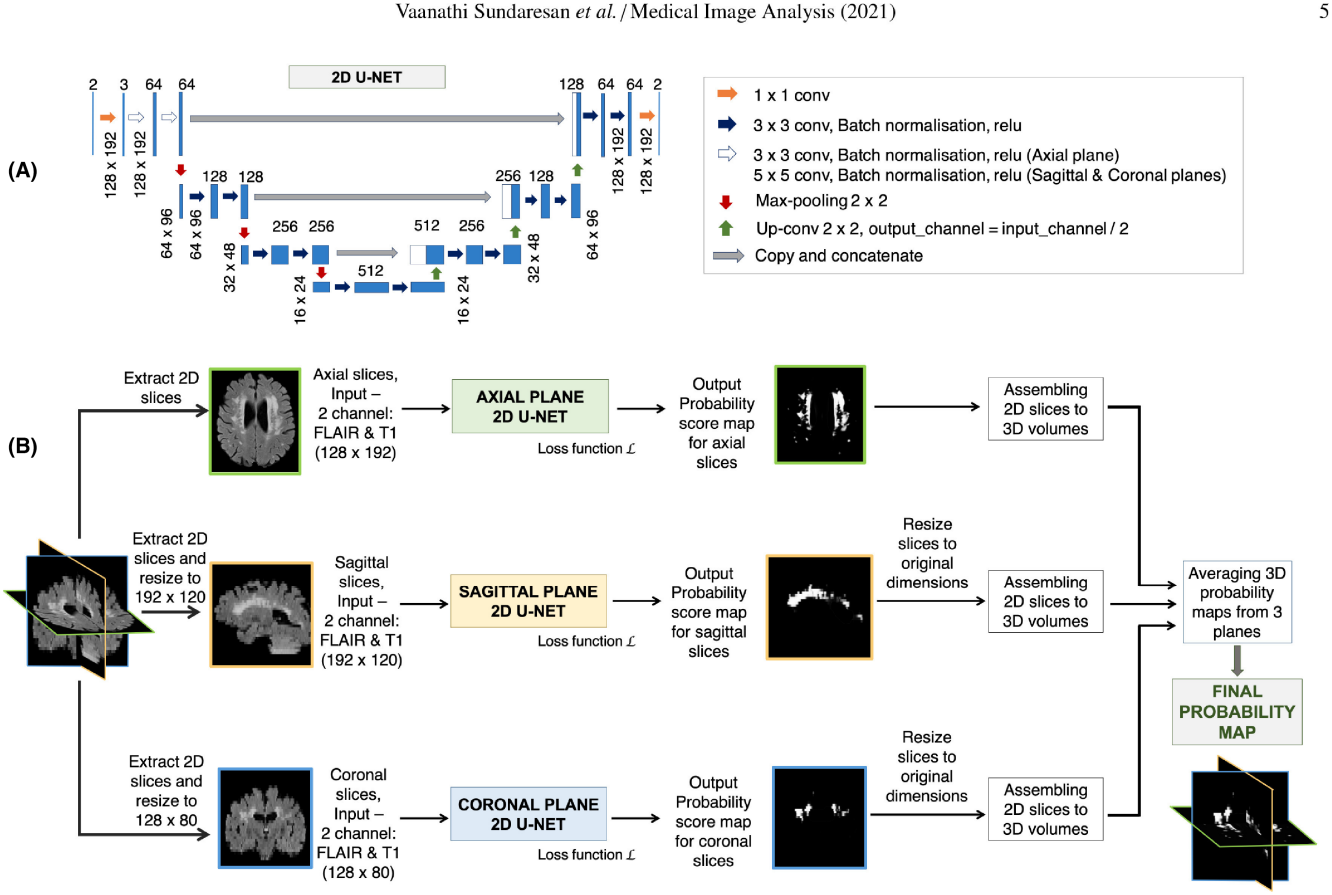
Triplanar U‐Net ensemble network (TrUE‐Net) used for segmentation of WHM. Reprinted with permission from Sundaresan et al.^32^ WHM, vascular white matter hyperintensity.

The intelligent diagnosis of WMH first obtains the ROI of WMH through the segmentation network, then classifies the WMH based on the Fazekas scale evaluation rules using a classification network, and finally obtains the WMH score for clinical diagnosis. Research on WMH segmentation based on deep learning networks has always been a hotspot of CSVD^26^, ^27^, ^28^, ^29^, ^30^, ^31^, ^32^, ^33^, ^34^, ^35^, ^36^ (Figure 4), and in view of the difficulty of WMH segmentation, many studies have made improvements based on traditional segmentation algorithms. For example, Ghafoorian et al.^21^ incorporated anatomical location information into a deep CNN and constructed a location‐sensitive deep CNN network to segment WMH regions. The results for the test dataset with 50 cases showed that the model had an average Dice of 0.792, which is comparable to the result of manual delineation (Dice = 0.805). Furthermore, the assessment of the progression of WMH has been a challenging task due to factors, such as genetics, age, and physical status (e.g., hypertension, smoking, and stroke). Rachmadi et al.^37^ constructed an end‐to‐end WMH progression prediction model using a deep CNN. By inputting the original T2‐FLAIR image and WMH mask, the Fazekas of each layer, which is in the image under different tasks (such as DWMH or PVH), and the image layer index corresponding to the maximum score can be obtained. This model could automatically predict and estimate the dynamic changes in WMH in space from the baseline to the next time point, and the average prediction accuracy of degradation/progress reached 74.74%. Accurate prediction of WMH progression is of great significance for assisting the clinical diagnosis of CSVD, and related prediction models need to be further developed.

### 3.4 Application of AI in CMBs

CMBs are small lesions resulting from the extravasation of blood through the vessel wall due to severe damage to the small vessel wall, with hemosiderin deposition as the main histological feature. The high prevalence areas are primarily localized in the temporo‐occipital region, the outer, upper, and posterior thalamus of the basal ganglia region, and the corticomedullary junction of the basal ganglia region.^9^ In T2*‐weighted gradient‐recalled echo (GRE) or susceptibility‐weighted imaging (SWI) images, CMBs generally appear as uniform round or oval low‐signal areas with diameters of 2–5 mm, with a maximum of 10 mm, and no edema is present around them. They cannot be directly observed in other traditional MRI images. In clinical practice, the number of CMBs is an independent predictor of the severity of cognitive impairment. The number of CMBs ≥3 is generally considered to be related to dementia and vascular dementia.^9^

AI‐based diagnostic methods mostly use segmentation to count and evaluate CMBs.^21^, ^38^ For example, Duan et al.^20^ constructed a segmentation model based on a dataset of 359 cases to segment CMBs. They achieved a dice value of 0.503 in the test dataset, which is comparable to the delineation results of three 3‐year‐old physicians (0.456, 0.514, and 0.549, respectively); however, the segmentation speed was 100 times faster than manual delineation. In the VALDO challenge, nine teams engaged in the detection and segmentation of cerebral microbleeds, utilizing a combined dataset of T2, T2*, and T1‐weighted images in T2* space from 219 patients, encompassing 1681 lesions. The methodologies implemented included UNet, nnUnet, a combination of UNet and Mask R‐CNN, as well as UNet combined with ResNet. Performance evaluation revealed a detection F1 score of 68.42, and a mean Dice score for segmentation of 84.01.^22^

### 3.5 Application of AI in PVS

PVSs are fluid‐filled areas that surround the brain arteries or veins and increase with age. PVSs have similar characteristics to cerebrospinal fluid in all MRI sequences, with linear, oval, or circular hyperintense signals in T2‐weighted imaging. Many studies have demonstrated that PVSs can reflect the presence of CSVD and are potential biomarkers of brain diseases, such as Alzheimer's disease, stroke, multiple sclerosis, and Parkinson's disease.^39^ In traditional clinical diagnosis, clinicians comprehensively consider the size, count, and distribution of PVSs in the brain to assess the risk level of patients with CSVD‐related diseases.^9^ The current PVS assessment mainly relies on manual visual assessment methods that are searched from images, but due to the progressive increase of PVSs, which are widely present in the brain, and the many structures similar to those in MRI images (such as cerebrospinal fluid), manual labeling of individual PVSs is challenging and time‐consuming. Therefore, a more objective, rapid, and automatic assessment method than manual visual assessment is urgently required.

Currently, the application of AI in PVS quantitation is primarily based on detection networks.^40^, ^41^, ^42^ In the deep detection network framework, the feature semantic information of the underlying network is less, but the location information is more accurate, whereas the feature semantic information of the high‐level network is richer but the location information is relatively rough. Traditional detection algorithms, such as Fast R‐CNN^43^ and Mask‐RCNN^23^ are based on high‐level network features, that is, the feature map of the last layer of the network for detection. These methods are more suitable for single‐scale object detection tasks and are not sufficiently friendly for object detection tasks involving large‐scale variations. Lin et al.^40^ proposed a feature pyramid network that integrates high‐resolution location information of low‐level features and semantic information of high‐level features to effectively solve the shortcomings of multi‐scale object detection tasks. Therefore, it is more suitable for PVS detection at different scales.

In addition, the clinical assessment of PVS mostly quantifies the count of PVS lesions in different brain regions (such as the basal ganglia, midbrain, and center of the semiovale), and the PVS burden cannot be regarded as a continuous rather than a standard measurement method. The continuous assessment method can better isolate normal brain structural changes during aging from the pathological burden of PVS. Dubost et al.^41^ proposed a CNN model for T2‐weighted imaging of 2115 subjects and calculated the count of PVS in the midbrain, hippocampus, basal ganglia, and centrum. The manual visual scores and the model scores for these four regions were consistent, with intraclass correlation coefficients (ICCs) between 0.75 and 0.88. The results suggest that this approach has the potential to replace manual visual scoring, thereby facilitating large‐scale epidemiological and clinical studies on PVS. In the VALDO challenge, four teams focused on the detection and segmentation of PVS using a dataset comprising T1‐weighted, T2‐weighted, and FLAIR images from 106 patients, encompassing a total of 34,328 lesions. The methods employed included nnUNet, Mask R‐CNN, UNet, and Random Forest (RF). The performance of these methods was quantified using two metrics: for detection, an F1 score of 38.92 was achieved, while for segmentation, the mean Dice score was 72.38.^22^

### 3.6 Application of AI in brain atrophy

Brain atrophy refers to the phenomenon of reduced brain volume unrelated to specific, grossly focal injuries, such as trauma and cerebral infarction. It is mainly manifested by widening of the cerebral sulcus, enlargement of the cerebral cisterns and/or ventricles, thinning of the cortex, and reduction of the white matter volume. Cortical atrophy is manifested by the deepening of cerebral sulci and widening of cerebral fissures, while medullary atrophy is evidenced by ventricular enlargement, and generalized brain atrophy is distinguished by widening of the sulci, cisterns, and ventricles. According to the extent of involvement, the following categories can be identified: (1) extensive cerebral atrophy, mainly including cerebral cortex and medullary types, and (2) localized brain atrophy, mainly confined to specific brain lobes, brain regions, one side of the brain, the cerebellum, the brain stem, and the hippocampus.^44^ The visual method can be used for simple evaluation, and visual semi‐quantitative evaluation can also be carried out by combining linear measurements on MRI images. Precise measurement can also be conducted using volume measurements obtained from software based on the three‐dimensional volume imaging of the brain, and the specific brain area measured should be indicated in the description. Clinically, the more commonly used ones are the medial temporal lobe atrophy (MTA) score, global cortical atrophy (GCA) score, and parietal lobe atrophy scale (Koedam score).^45^, ^46^, ^47^

**FIGURE 5:**
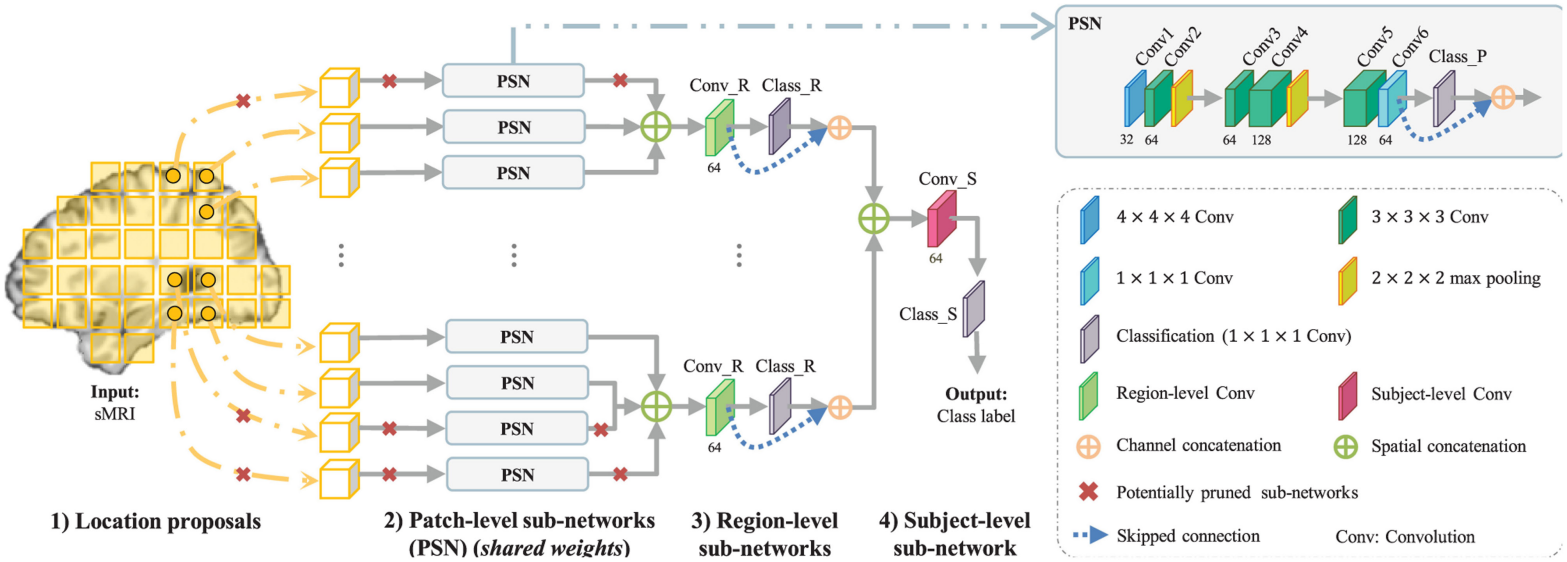
Hierarchical fully convolutional network (H‐FCN) used for the location of brain atrophy. Reprinted with permission from Lian et al.^56^

Brain atrophy quantification is fundamental to the study of brain development, neurological disorders, and neuroinformatics and is one of the predictors of cognitive impairment in patients with CSVD. Quantitative assessment is performed by manually delineating areas of brain atrophy on MRI; however, it is time‐consuming and labor‐intensive, and the accuracy of longitudinal brain atrophy quantification methods cannot be tested. Therefore, deep learning has also been explored to address this problem^34^, ^48^, ^49^ (Figure 5). Bernal et al.^50^ proposed a deep learning framework to generate longitudinal datasets by deforming T1‐weighted brain MRI scans as requested through segmentation maps. The model incorporates a cascaded multi‐path U‐Net optimized with a multi‐objective loss that allows its paths to generate different brain regions accurately, and it was observed that the framework could produce synthetic follow‐up scans that matched the real ones (total scans = 584; median absolute error: 0.03 ± 0.02; structural similarity index: 0.98 ± 0.02; Dice similarity coefficient: 0.95 ± 0.02; percentage of brain volume change: 0.24 ± 0.16; Jacobian integration: 1.13 ± 0.05). It is important to study longitudinal changes in brain atrophy.

### 3.7 Application of AI in other MRI features of CSVD

Following the definitive characterization of cortical superficial siderosis and cortical cerebral microinfarcts as MRI features of CSVD in 2023, as delineated in the STRIVE guidelines,^11^ the application of AI in analyzing these two MRI features remains relatively limited. This gap is primarily due to the recent formal recognition of these features, which has led to a nascent stage of research in AI applications focused on their detection and analysis. Consequently, further development and refinement in AI methodologies specifically targeting these newly defined CSVD markers are necessary to enhance diagnostic accuracy and understanding in the field.^11^

## 4 CONCLUSIONS

This paper summarized the published literature on the applications of AI to MRI of CSVD, mainly including the application of CNNs in the analysis of typical imaging of CSVD, such as RSSI, lacunes of premed vascular origin, WMH, PVS, CMBs, and brain atrophy.

In general, little research on AI in CSVD exists at present and has mainly been conducted in the past 3 years, indicating that this research direction is relatively new. Second, most of the existing studies focus on the segmentation of lesions, which shows that the size and number of lesions play important roles in the diagnosis of CSVD in clinical practice. Simultaneously, the results show that there is more research on AI based on WMH and brain atrophy, which is the current research hotspot. This situation may be present because both diseases have high incidence rates in clinical practice. WMH can be diagnosed by one or two simple sequences, such as T2‐weighted imaging and T2‐FLAIR, and objective quantitative evaluation criteria exist for WMH, which are simple and easy to implement. Brain atrophy is considered an independent factor for predicting the decline of cognitive function and has strong visibility and measurability in imaging. Some researchers and clinicians have taken brain atrophy as an imaging marker to evaluate the progress or treatment effect of CSVD. However, with the in‐depth study of CSVD, the guiding significance of other signs for clinical intervention has been increasingly reflected. For example, when the number of CMBs in the brain is ≥5, the risk of cerebral hemorrhage and death after cerebral hemorrhage may exceed the benefits of antithrombotic drugs, as the use of antithrombotic drugs for such patients has unpredictable risks, and intelligent analysis based on big data may play an important role in this direction.^51^

**FIGURE 6:**
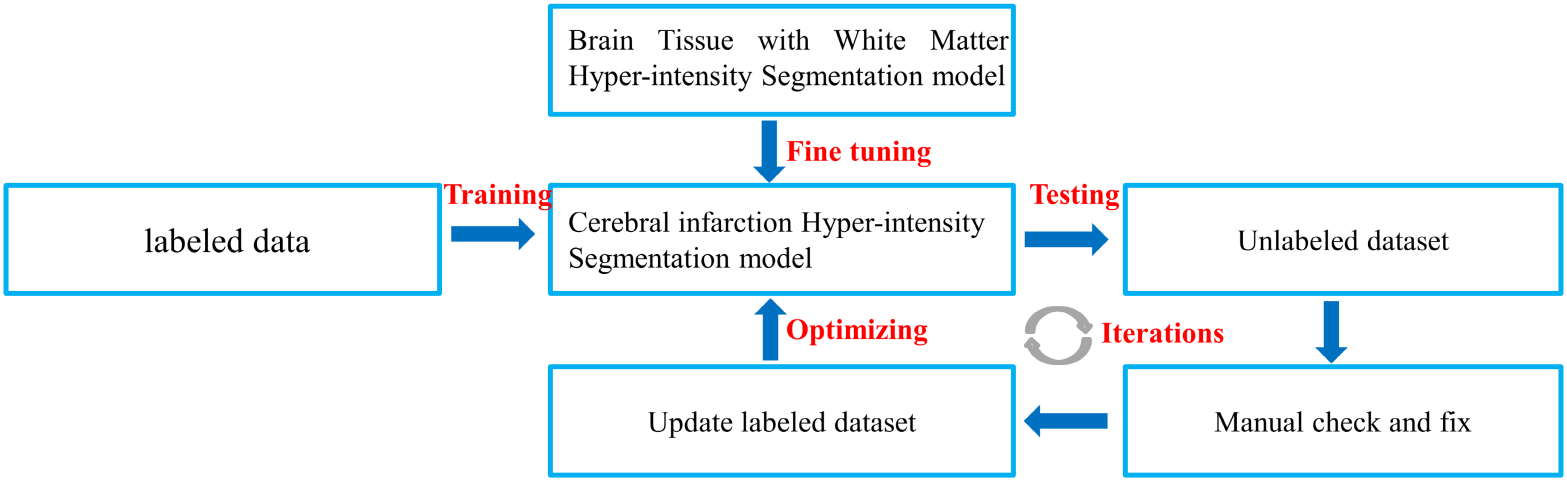
Flowchart of deep learning network optimization based on transfer learning.

Sufficient and multi‐center datasets are the basis for obtaining a robust and high‐performance model. Especially for clinical applications, obtaining more accurate results is important. Therefore, in the existing research, multi‐center data and public datasets are used to train and test the algorithm, which makes the results of the model reliable. However, in order to apply the model to clinical practice, the model needs to be tested on a larger scale of multi‐center data. Therefore, effective algorithm optimization methods require more research, such as the use of transfer learning and the human‐in‐the‐loop method to integrate the network parameters of the existing models with prior knowledge to improve the performance of AI algorithms, as shown in Figure 6. Simultaneously, the disclosure of medical data is also a key factor in accelerating the rapid clinical transformation of AI.

The rapid advancement in the application of large language models (LLMs) in medical image analysis, notably in understanding and processing medical images, exhibits vast potential. Wang et al. introduced an approach integrating LLMs into medical imaging CAD networks.^52^ This method synergizes the LLMs' domain knowledge in medicine and logical reasoning with the visual understanding capabilities of medical imaging CAD models by summarizing and reorganizing information in a natural language text format. Another significant development is “MedSAM,” a deep learning model for universal medical image segmentation, building on a dataset of over one million medical image‐mask pairs, MedSAM adapts the segment anything model (SAM) for medical purposes, showcasing superior performance over existing segmentation models, underscoring its potential in clinical and research applications.^53^ LLMs are poised to significantly influence CSVD MRI feature recognition and analysis. Multimodal LLMs, integrating visual and textual data, offer enhanced accuracy in identifying and interpreting CSVD MRI features, and process both images and medical documentation for comprehensive diagnostics. Advancements in deep learning models for image segmentation, particularly for CSVD characteristics like white matter hyperintensities, lacunes, and microbleeds, are aiding early diagnosis and monitoring of CSVD progression. Beyond diagnostic accuracy, LLMs are increasingly applied in clinical settings for disease monitoring and management, such as analyzing MRI time‐series data to track CSVD progression and assess treatments. These developments are crucial in advancing clinical research and streamlining medical image data processing, thereby improving efficiency and outcomes in healthcare.

Through the continuous improvement of AI, with the development of high‐performance parallel computing technology and improved quality of medical images, deep learning‐based medical image analysis will achieve wider application and progress. We firmly believe that, after sufficient and effective model training, an AI algorithm comparable to that of a doctor with a certain seniority can be obtained, which will surely become a powerful tool to assist doctors in clinical diagnosis. However, it should be emphasized that the purpose of AI is not to replace doctors but to assist doctors in making better clinical diagnoses. Additionally, although AI technology has a positive impact on medical image analysis, several challenges remain to be addressed. (1) CSVD appears as small pixel‐level lesions on MRI images, which usually consist of only a few pixels. The precise quantitative evaluation of such lesions poses a huge challenge to the accuracy of the algorithm. (2) Comprehensive evaluation of different magnetic resonance instruments and multiple sequences, combined detection, and quantitative evaluation of six types of CSVD imaging features with different incidence rates, disease locations, and sizes are the keys to clinical diagnosis. However, how to embed CSVD is still a lack of research on the intelligent diagnosis and treatment process. (3) In addition to image data, according to the historical medical data, subject behaviors, biochemical tests of patients, etc., it is also possible to evaluate the brain health status of subjects and obtain hints on the potential risk of cerebrovascular diseases, so as to assist doctors in diagnosing and evaluating the brain health and nervous system problems of the subjects. However, such combined research is still rare at present. (4) The application of AI is characterized as a black box; therefore, explaining how it works is difficult without understanding the internal representation. How to combine clinical practice more effectively to provide doctors with an AI diagnosis basis and help doctors explain clinical problems is also a question worthy of discussion. It is believed that with the establishment of high‐quality databases and the continuous updating of related algorithms, AI will become a powerful tool to assist doctors in the clinical evaluation of CSVD and further cooperate with clinical practice to improve the accuracy of diagnosis and prognosis.

## FUNDING INFORMATION

This study has received funding from the National Natural Science Foundation of Chongqing (cstc2021jcyj‐msxm3744), Beijing Postdoctoral Research Funding Project (2023‐ZZ‐002), and China Postdoctoral Science Foundation (2023M742438).

## CONFLICT OF INTEREST STATEMENT

The authors of this manuscript declare no relationships with any companies, whose products or services may be related to the subject matter of the article.

## Data Availability

The datasets generated during and/or analyzed during the current study are available from the corresponding author on reasonable request.
